# Clonal Expansion of a *Streptococcus pneumoniae* Serotype 3 Capsule Variant Sequence Type 700 With Enhanced Vaccine Escape Potential After 13-Valent Pneumococcal Conjugate Vaccine Introduction

**DOI:** 10.1093/infdis/jiae040

**Published:** 2024-03-26

**Authors:** Akuzike Kalizang'oma, Todd D Swarthout, Thandie S Mwalukomo, Arox Kamng’ona, Comfort Brown, Jacquline Msefula, Hayley Demetriou, Jia Mun Chan, Lucy Roalfe, Uri Obolski, Jose Lourenço, David Goldblatt, Chrispin Chaguza, Neil French, Robert S Heyderman

**Affiliations:** NIHR Mucosal Pathogens Research Unit, Research Department of Infection, Division of Infection and Immunity, University College London, London, United Kingdom; Pneumonia and Meningitis Pathogens Associate Research Group, Malawi-Liverpool-Wellcome Research Programme, Blantyre, Malawi; NIHR Mucosal Pathogens Research Unit, Research Department of Infection, Division of Infection and Immunity, University College London, London, United Kingdom; Pneumonia and Meningitis Pathogens Associate Research Group, Malawi-Liverpool-Wellcome Research Programme, Blantyre, Malawi; Julius Center for Health Sciences and Primary Care, University Medical Center Utrecht, Utrecht University, Utrecht, Netherlands; School of Medicine and Oral Health, Kamuzu University of Health Sciences, Blantyre, Malawi; School of Life Sciences and Allied Health Professionals, Kamuzu University of Health Sciences, Blantyre, Malawi; Pneumonia and Meningitis Pathogens Associate Research Group, Malawi-Liverpool-Wellcome Research Programme, Blantyre, Malawi; Pneumonia and Meningitis Pathogens Associate Research Group, Malawi-Liverpool-Wellcome Research Programme, Blantyre, Malawi; Great Ormond Street Institute of Child Health, University College London, London, United Kingdom; NIHR Mucosal Pathogens Research Unit, Research Department of Infection, Division of Infection and Immunity, University College London, London, United Kingdom; Great Ormond Street Institute of Child Health, University College London, London, United Kingdom; Porter School of the Environment and Earth Science, Tel-Aviv University, Tel-Aviv, Israel; Faculdade de Ciências, BioISI, Universidade de Lisboa, Lisbon, Portugal; NIHR Mucosal Pathogens Research Unit, Research Department of Infection, Division of Infection and Immunity, University College London, London, United Kingdom; Great Ormond Street Institute of Child Health, University College London, London, United Kingdom; NIHR Mucosal Pathogens Research Unit, Research Department of Infection, Division of Infection and Immunity, University College London, London, United Kingdom; Parasites and Microbes Programme, Wellcome Sanger Institute, Hinxton, United Kingdom; Department of Epidemiology of Microbial Diseases, Yale School of Public Health, Yale University, New Haven, Connecticut, USA; Yale Institute for Global Health, Yale University, New Haven, Connecticut, USA; Institute of Infection, Veterinary and Ecological Sciences, Department of Clinical Infection, Microbiology and Immunology, University of Liverpool, Liverpool, United Kingdom; NIHR Mucosal Pathogens Research Unit, Research Department of Infection, Division of Infection and Immunity, University College London, London, United Kingdom; Pneumonia and Meningitis Pathogens Associate Research Group, Malawi-Liverpool-Wellcome Research Programme, Blantyre, Malawi

**Keywords:** *Streptococcus pneumoniae*, serotype 3, pneumococcal conjugate vaccine, capsule, Africa

## Abstract

**Background:**

*Streptococcus pneumoniae* serotype 3 remains a problem globally. Malawi introduced 13-valent pneumococcal conjugate vaccine (PCV13) in 2011, but there has been no direct protection against serotype 3 carriage. We explored whether vaccine escape by serotype 3 is due to clonal expansion of a lineage with a competitive advantage.

**Methods:**

The distribution of serotype 3 Global Pneumococcal Sequence Clusters (GPSCs) and sequence types (STs) globally was assessed using sequences from the Global Pneumococcal Sequencing Project. Whole-genome sequences of 135 serotype 3 carriage isolates from Blantyre, Malawi (2015–2019) were analyzed. Comparative analysis of the capsule locus, entire genomes, antimicrobial resistance, and phylogenetic reconstructions were undertaken. Opsonophagocytosis was evaluated using serum samples from vaccinated adults and children.

**Results:**

Serotype 3 GPSC10-ST700 isolates were most prominent in Malawi. Compared with the prototypical serotype 3 capsular polysaccharide locus sequence, 6 genes are absent, with retention of capsule polysaccharide biosynthesis. This lineage is characterized by increased antimicrobial resistance and lower susceptibility to opsonophagocytic killing.

**Conclusions:**

A serotype 3 variant in Malawi has genotypic and phenotypic characteristics that could enhance vaccine escape and clonal expansion after post-PCV13 introduction. Genomic surveillance among high-burden populations is essential to improve the effectiveness of next-generation pneumococcal vaccines.

## Teaser

A ST700-GPSC10 serotype 3 *Streptococcus pneumoniae* lineage, currently restricted to the African continent, is characterized by the absence of 6 genes from the capsular polysaccharide locus and decreased susceptibility to opsonophagocytosis, which may explain the spread of this lineage.

*Streptococcus pneumoniae,* an encapsulated bacteria that commonly colonizes the human nasopharynx, is responsible for >300 000 deaths annually worldwide, with the greatest burden in low- and middle-income countries [1, 2]. The pneumococcus has >100 biochemically and antigenically distinct capsule types, with 20–30 types accounting for most cases of invasive pneumococcal disease (IPD) [3]. Serotype 3 pneumococci have unique genetic, phenotypic, and epidemiologic characteristics [4], including a thicker capsule that is more loosely attached to the bacterial surface. In a murine model, it has been shown that serotype 3 capsular polysaccharides (CPSs), when released from the bacterial surface, uniquely interfere with antibody-mediated bacterial killing and protection [5]. Although there is evidence of direct (vaccine-induced) protection by the widely introduced 13-valent pneumococcal conjugate vaccine (PCV13) against *S. pneumoniae* serotype 3 IPD in children and adults, studies from Denmark, France, Greece, Portugal, Sweden, the United Kingdom, and the United States show limited or no effectiveness of PCV13 against serotype 3 pneumococcal carriage [6, 7].

Lineages of serotype 3 vary substantially with geographic location, and they show a tendency to recombine and segregate greater than other serotypes [8, 9]. The clonal complex 180 (CC180), which corresponds to the GPSC12 lineage based on the Global Pneumococcal Sequence Cluster (GPSC) nomenclature [10], termed GPSC12-CC180, is the most dominant serotype 3 strain worldwide comprising 3 lineages (Iα, Iβ, and II) [8]. Compared with other GPSC12-CC180 lineages, the clade II lineage is characterized by a higher prevalence of antimicrobial resistance (AMR), greater diversity of surface protein antigens, and a higher rate of recombination mediated by transposons, phages, and integrative conjugative elements carrying genes associated with competence and virulence [8].

Malawi introduced PCV13 into the national Expanded Programme on Immunisation in 2011, using a 3 + 0 schedule (1 dose at 6, 10, and 14 weeks of age) with a catch-up campaign limited to children aged <1 year. There has been high PCV13 coverage, exceeding 90%, with direct protection against vaccine serotype IPD among vaccinated infants [11]. However, in line with reports from Kenya and The Gambia [12, 13], indirect protection of older unvaccinated children and adults has been less effective than in high-income countries, with relatively high residual carriage of all 13 vaccine serotypes among PCV13-vaccinated children, up to 8 years after PCV13 introduction in Malawi [1, 14]. In particular, there has been no apparent direct protection against serotype 3 carriage [14], and population-level serosurveillance suggests limited vaccine-induced serotype 3–specific immunity [15]. Because severe disease is a dead end for *S. pneumoniae*, virulence and pathogenicity largely develop as a consequence of active pneumococcal adaptation to colonize the host and transmit from person to person. We therefore hypothesized that the apparent vaccine escape by serotype 3 in Malawi could be due, at least in part, to the clonal expansion of a lineage with a competitive advantage in the nasopharyngeal niche.

Through an analysis of the genome sequences of 135 serotype 3 isolates from sequential carriage surveys [14, 16], we describe the clonal expansion of a sequence type (ST) 700–GPSC10 serotype 3 lineage in Malawi following PCV13 introduction, a lineage distinct from the GPSC12-CC180 lineage described elsewhere [8]. ST700-GPSC10 is characterized by the absence of 6 genes in its serotype 3 CPS locus, a different AMR profile from other serotype 3 lineages in Malawi, and lower susceptibility to opsonophagocytosis that may explain the spread of this lineage with enhanced evasion of vaccine-induced immunity.

## METHODS

### Genome Sequences

The genome sequences of 135 serotype 3 carriage isolates were used in this analysis (Supplementary Table 1) from pneumococcal carriage surveillance between 2015 and 2019 in Blantyre, Malawi [14, 16]. An additional 592 genome sequences were accessed from the Global Pneumococcal Sequencing (GPS) project spanning >2 decades (1993–2018) in 32 countries worldwide, including Malawi [10, 17].

### Genome Analysis

GPSCs and STs based on the pneumococcal multilocus sequence typing scheme [18] were determined using PopPUNK software (version 2.6.0) [19] and PubMLST, respectively [20]. We scanned each genome for the presence of AMR genes (*tetM, cat, mefA,* and *ermB*), using Abricate software (version 1.0.1) with the ResFinder database (https://github.com/tseemann/abricate) and PathogenWatch (https://pathogen.watch/) with ResFinder and the National Center for Biotechnology Information databases; minimum inhibitory concentration (MICs) to penicillin were genotypically predicted using an analysis pipeline developed by the US Centers for Disease Control and Prevention [21]. The MICs were interpreted using Clinical and Laboratory Standards Institute guidelines and clinical breakpoints (https://clsi.org/). Genome assemblies were generated using SPAdes software (version 2.5) [22]. CONTIGuator software (version 2.0) [23] was used to order the contigs in the assemblies of the Malawian serotype 3 isolates, and serotype 3 (strain 670-6B; GenBank accession no. NC_014498) was used as a reference.

The CPS locus is known to be physically located between *aliA* and *dexB* genes in the pneumococcus [24]. The prototypical serotype 3 CPS reference locus [24] has the following genes, *dexB, aliB, tnp, wzg, wzh, wzd, wze, tnp, ugd, wchE, galU,* and *pgm*, and our analysis is based on the gene content comparisons at the physical locations with the serotype 3 reference CPS locus. Although only the core CPS genes (*wzg, wzh, wzd,* and *wze*) have known capsule biosynthesis functions [24, 25], others have unknown or potential auxiliary functions. Therefore, to avoid confusion, we refer to any genes located in the entire region known to harbor genes involved in capsule biosynthesis for serotype 3 as *CPS genes*. To locate the CPS locus and manually extract the corresponding sequences, each ordered genome was first compared against the serotype 3 reference genome described by Bentley et al [24] using blastn software (version 2.14.0) [26] and the Artemis Comparison Tool (version 18.0.3) [25]. The presence of serotype 3 capsule locus genes was also assessed by aligning the sequencing reads of each strain to the sequence of the CPS locus described by Bentley et al in 2006 [24] (using Bowtie2 [27] and Samtools [28] software).

The presence and absence of the CPS locus genes was done by mapping each genome against the serotype 3 reference CPS locus sequence (GenBank accession no. CR931634) from Bentley et al [24]. We used Snippy software (version 4.3.6) to map reads of each isolate against the reference CPS locus sequences to identify missing genes. The contiguity of genes present was assessed using the Artemis Comparison Tool (version 18.1.0) [25]. Genome comparisons between different serotype 3 lineages were carried out using whole-genome alignment and maximum-likelihood was estimated [27, 28]. The phylogeny was assessed in a subset of isolates (RaxML [29]; GTRGAMMA, 100 iterations), which included all 135 serotype 3 isolates from Malawi [14], as well as all 18 non–serotype 3 strains belonging to GPSC10 isolated in the same Malawi study [14] (Supplementary Table 1). Graphic and statistical analysis was carried out using R software (version 3.2; https://www.R-project.org/).

### Bacterial Growth

Representative strains from ST700 and ST5435 were grown overnight on Columbia blood agar supplemented with 5% horse blood at 37°C and 5% carbon dioxide (microaerophilic condition) or at 37°C anaerobically using the AnaeroGen W-Zip Compact system (Oxoid), per the manufacturer's instructions. Image brightness and contrast were adjusted using Fiji software (version 2.14.0).

### Opsonophagocytosis Assay

We undertook opsonophagocytosis analysis to evaluate bacterial resistance to antibody plus complement mediated killing, as described elsewhere [30]. Briefly, 3 ST700 and 3 ST5435 serotype 3 strains were selected at random from the isolate collection. These were compared to killing reduction against a reference serotype 3 strain (an optochin resistant variant of Wu2), in serum samples from 5 healthy adults vaccinated with 23-valent pneumococcal polysaccharide vaccine and from 6 PCV13-vaccinated children. Todd-Hewitt broth with yeast extract and filtered fetal bovine serum (4%) were used for the assay. The incubations were carried out in a 96-well plate with serial dilutions of human serum before further incubation with HL-60 cells and baby rabbit complement. Wells were plated out onto agar, and bacterial colonies were quantified with a plate reader. Opsonophagocytosis titers were expressed as the opsonic index [30].

### Statistical Analysis

Statistical tests and associated diagrams were generated using R software (version 2.11.1) (R Core Team 2014; https://www.R-project.org/) and GraphPad Prism (version 8.0) (GraphPad Software). Nonparametric data, which included opsonic indices, are presented as individual data points and median values, and Mann-Whitney tests were used to compare theses indices. Differences were considered significant at *P* < .05.

### Data Availability

Pneumococcal CPS locus de novo assemblies for representative STs are available from the National Center for Biotechnology Information (accession nos. OR805039–OR805049).

## RESULTS

### GPSC10-ST700 as the Most Prominent *S. pneumoniae* Serotype 3 Genotype in Malawi

**Figure 1.**
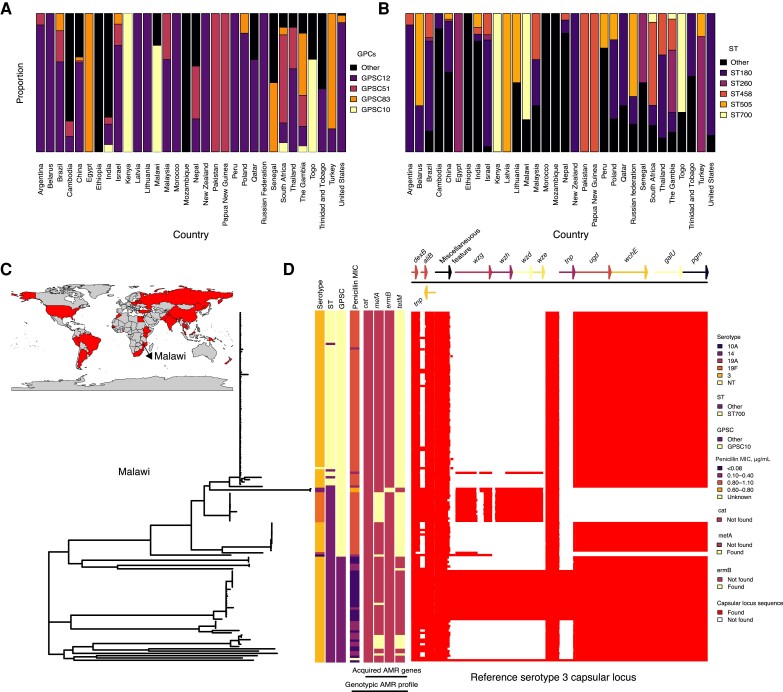
Countries of origin and genomic profiles of *Streptococcus pneumoniae* serotype 3 isolates. *A,* Global Pneumococcal Sequence Clusters (GPSCs) isolated in each country. *B*, Sequence types (STs) isolated in each country. *C*, Countries (*in red*) from which the serotype 3 *S. pneumoniae* isolates described in this analysis originated. *D*, Maximum-likelihood tree including all serotype 3 pneumococci isolated from Blantyre, Malawi [14], and the non–serotype 3 strains belonging to the GPSC10 lineage. The tree is annotated with metadata columns showing (from left to right) the serotype, ST, GPSC, penicillin minimum inhibitory concentration (MIC) (predicted genetically), the presence and absence of antimicrobial resistance (AMR) genes (*cat, mefA, ermB, tetM*), and the presence and absence of each gene annotated on the serotype 3 capsular polysaccharide (CPS) locus [24]. In white are the absent 6 genes, and in red are the core CPS-3 genes (in reference to the serotype 3 locus defined by Bentley et al [24], shown above the gene locus panel).

We first compiled a global collection of whole-genome–sequenced serotype 3 isolates from the GPS data set to understand the geographic distribution of GPSC10-ST700. The carriage and invasive isolates included in this analysis originated from 32 countries worldwide, including Malawi. The proportion of the most common serotype 3 GPSCs and STs within each country is shown in Figure 1*A* and 1*B* (detailed in Supplementary Table 1) and displayed geographically in Figure 1*C*. Among the sequenced genomes, we identified serotype 3 GPSC10 in 6 countries (Figure 1*A*–1*C*): Kenya (14 of 14 isolates), India (1 of 20), Malawi (33 of 43), South Africa (7 of 110), The Gambia (2 of 49), and Togo (4 of 6). In contrast, although as reported previously [8], most of the serotype 3 isolates globally were associated with the GPSC12 lineage, including strains from the CC180 clone, we did not find the GPSC12 lineage among the 135 serotype 3 carriage isolates from Malawi or in our previous genomic analysis of invasive and carriage isolates from the same population [31, 32]. Instead, among the 135 carriage serotype 3 isolates from Malawi collected after the introduction of PCV13, GPSC10-ST700 was most prominent (72 of 135 isolates). Pneumococcal carriage studies have shown that serotype 3 has expanded after PCV13 introduction in Malawi [14], with ST700 now the most prominent clone [10].

### Multiple Absent Genes in the CPS Locus of GPSC10-ST700

We then assessed the genetic diversity of the serotype 3 isolates from Malawi to understand the genetic attributes of GPSC10-ST700 serotype 3 isolates that may have contributed to their dominance compared with the other serotype 3 lineages in Malawi. First, mapping the sequences of each serotype 3 isolate genome against the prototypical serotype 3 reference CPS locus sequence described by Bentley et al [24], we observed an absence of 6 genes in the CPS locus of the GPSC10-ST700 lineage (Figure 1*D* and Table 1). We confirmed this finding after performing de novo assemblies of the whole-genome sequences to reconstruct the CPS locus. We considered all the genes located between *dexB* and *pgm* in the serotype 3 reference CPS locus sequence described by Bentley et al [24] as part of the CPS locus (see Methods). We found that this CPS locus variant characterized by the absent genes was not exclusive to the GPSC10-ST700 lineage; rather, it is polyphyletic, appearing independently in ≥3 distinct phylogenetic clusters expressing serotype 3, including ST700 and ST3214. Despite the absent genes in the CPS locus, the resulting shorter CPS locus sequence still included the core CPS genes (*wchE*, *ugd*, and *galU*) necessary to produce the serotype 3 polysaccharide [24].

**Table 1.** Details of the 6 Absent Genes in the Sequence Type 700 Serotype 3 Capsular Polysaccharide Locus^a^

Feature Name	Locus Tag	Start	End	Feature Length	Feature Product
*aliB*	SPC03_0002	321	494	174	Putative oligopeptide-binding protein AliB (pseudogene)
*tnp*	SPC03_0003	487	1336	850	Putative IS630-Spn1 transposase
*wzg* (*cpsA*)	SPC03_0004	1524	2748	1225	Integral membrane regulatory protein Wzg (pseudogene)
*wzh* (*cpsB*)	SPC03_0005	2750	3480	731	Protein-tyrosine phosphatase Wzh (pseudogene)
*wzd* (*cpsC*)	SPC03_0006	3489	4181	693	Capsular polysaccharide biosynthesis protein Wzd
*wze* (*cpsD*)	SPC03_0007	4191	4604	414	Tyrosine-protein kinase Wze (pseudogene)

^a^Indicated in blue in Figure 1*D* (capsular polysaccharide reference sequence described by Bentley et al [24]; GenBank accession no. CR931634).

**Figure 2.**
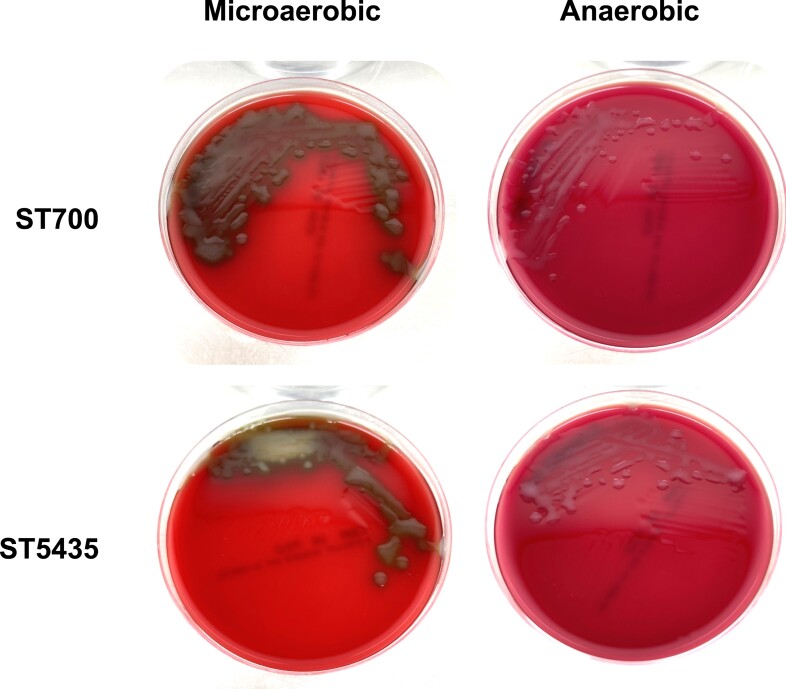
*Streptococcus pneumoniae* serotype 3 sequence type (ST) 700 and ST5435 strains grown in microaerophilic and anaerobic conditions. Representative strains from ST700 and ST5435 serotype 3 collection were grown overnight on Columbia blood agar with 5% horse blood, under microaerophilic and anaerobic conditions.

Our literature search revealed that the observed serotype 3 CPS locus variant associated with GPSC10-ST700 was analogous to a serotype 3 variant isolated from deceased chimpanzees in 2011 in Kenya [33]. Although the authors described the missing genes as pseudogenes, our annotation highlights their potential involvement in polysaccharide production and export (Table 1). The GPSC10-ST700 isolates from Malawi produce a functional serotype 3 polysaccharide capsule, as assessed by latex agglutination [34] and typical colony morphology (Figure 2). We observed mucoid colonies with no morphological differences between representative strains showing the full (ST5435) and the variant (ST700) CPS locus when grown anaerobically or in a microaerophilic environment, although colony size and alpha hemolysis were reduced for both strains in anaerobic conditions (Figure 2). Owing to the absent 6 genes, ST700 strains lack homologues of *cpsB* (*wzh*)*, cpsC* (*wzd*), and *cpsD* (*wze*), which encode for a bacterial tyrosine kinase system important for the regulation of capsule production in D39 derivative strains (serotype 2), particularly at the septum and in low oxygen conditions [3, 35, 36]. As only *wchE*, *ugd*, and *galU* are involved in CPS-3 synthesis [24], the role that nonessential genes such as *cpsB* play in regulating capsule thickness or function in serotype 3 remains to be determined.

### GPSC10-ST700 Characterized by AMR and a Unique Genetic Profile

Next, we assessed the distribution of the AMR and virulence genes in the GPSC10-ST700 and other lineages associated with serotype 3 isolates. Figure 1*C* shows the maximum-likelihood phylogeny of all Malawian serotype 3 isolates (multiple GPSCs) together with GPSC10 strains associated with other serotypes. All except the isolates belonging to GPSC9 are characterized by the 6-gene absence seen in the serotype 3 cluster described above. Only GPSC10-ST700 genomes were characterized by the presence of a tetracycline resistance gene and by a predicted MIC to penicillin >0.5 µg/mL (penicillin nonsusceptible).

The maximum-likelihood phylogeny of all pneumococcal isolates from Blantyre, Malawi, collected between 2015 and 2019, showed that GPSC10 included isolates belonging to serotype 3 and other serotypes, including 19A, 19F, and 14. This observation suggests that the GPSC10 lineage tends to undergo capsule switching. The GPSC10 serotype 3 and the other capsule-switched isolates—GPSC10-19A, GPSC10-19F, GPSC10-10A, and GPSC10-14—were distinguished by a minimum of 4599, 3264, 7774, and 6232 single-nucleotide polymorphisms, respectively. Therefore, considering that the serotype 3 isolates in the GPSC10-ST700 lineage differed by up to 7120 single-nucleotide polymorphisms (similar to the genetic divergence between serotype 3 and the other serotypes in the GPSC10 lineage), we suggest that GPSC10-ST700 serotype 3 strains may have originated through multiple capsular switching events from the same original genomic background.

To identify genotypic traits that explain the clonal expansion of serotype 3 GPSC10-ST700, full-genome comparison was carried out between isolates belonging to the different serotypes within GPSC10. This analysis highlighted 2 gene clusters present in ST700 serotype 3 strains (Table 2). The first cluster (here named the *dpp-ABC cluster*) carries a dipeptide permease (dpp) adenosine triphosphate–binding cassette (ABC) transporter, and the second cluster (the lantibiotic cluster) carries the genes for the production and metabolism of lantibiotics, a class of bacteriocins expressed by gram-positive bacteria [29].

**Table 2.** Gene Clusters Potentially Associated With Pathogenicity—dipeptide permease Adenosine Triphosphate–Binding Cassette Transporter Cluster and Lantibiotic Cluster Present in Sequence Type 700 Serotype 3 Isolates

Gene Cluster	Feature Name	Feature Length	Feature Product	Accession No.
dpp-ABC transporter cluster	*Cyd*	407	Cytidine deaminase	cl00269
	*pgpA*	488	Phosphatidylglycerophosphatase A (lipid transport and metabolism)	COG1267
	*shp3*	1409	Hypothetical protein	cl14647
	*gsiA*	1982	ABC-type glutathione transport system ATPase component	COG1123
	*dppC*	851	ABC-type dipeptide/oligopeptide/nickel transport system (permease)	COG1173
	*dppB*	950	ABC-type dipeptide/oligopeptide/nickel transport system (permease)	COG0601
	*dppA*	1628	ABC-type dipeptide/oligopeptide/nickel transport system (ATPase)	cd00995
	*nanM*	923	N-acetylneuraminate (sialic acid) epimerase	cl37290
Lantibiotic cluster	*lanC*	407	Lanthionine synthetase C–like domain associated with serine/threonine kinases	cd04791
	*ptrB*	488	Protease II	COG1770
	*mdlB*	1409	ABC-type multidrug transport system, ATPase and permease component, ABC-type bacteriocin transporter	COG1132
				cl31074
	*lolD*	1982	ABC domain of the transporters involved in the export of lipoprotein and macrolide, putative bacteriocin export ABC transporter, ABC-type antimicrobial peptide transport system	cd03255
	*lhp5*	851	Hypothetical protein	…^a^

Abbreviations: ABC, adenosine triphosphate–binding cassette; ATPase, adenosine triphosphatase; dpp, dipeptide permease.

^a^No conserved domains were found in the frame.

### Reduced Opsonophagocytic Killing of GPSC10-ST700 Strains Highlighted by Phenotypic Analysis

**Figure 3.**
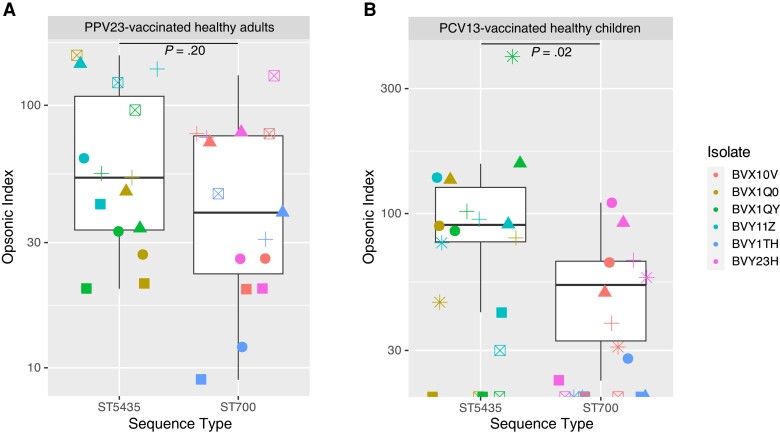
Opsonophagocytic killing of *Streptococcus pneumoniae* serotype 3 sequence type (ST) 700 and ST5435 isolates by serum samples from 5 healthy adults vaccinated with 23-valent pneumococcal polysaccharide vaccine (PPV23) (*A*) and 6 children vaccinated with 13-valent pneumococcal conjugate vaccine (PCV13) (*B*). Symbols differentiate serum samples from individual donors, and colors differentiate isolates.

Three serotype 3 ST700 isolates (CPS-3 gene cluster absent) and 3 serotype 3 ST5435 isolates (CPS-3 gene cluster present) from Blantyre, obtained between 2015 and 2019. Were assessed for their ability to survive opsonophagocytosis with immune serum samples. When tested both against serum samples from adults (vaccinated with 23-valent pneumococcal polysaccharide vaccine) and serum samples from children (PCV13 vaccinated), ST700 was less susceptible to opsonophagocytic killing than ST5435 (Figure 3). The median opsonic indices for ST700 and ST5435 were 39 and 53, respectively, with serum samples from adults (*P* = .20; Mann-Whitney test), and 25.5 and 83.5 with samples from children (*P* = .02; Mann-Whitney test).

## DISCUSSION

The control of *S. pneumoniae* serotype 3 carriage and disease has become a prominent challenge among vaccine developers and public health agencies [4, 14]. In contrast to other serotypes included in the PCV13 formulation, there is increasing evidence that PCV13 introduction has achieved no or only partial immunity to serotype 3 [4]. Moreover, as demonstrated by the CC180 lineage, serotype 3 has an increased tendency to recombine, which has resulted in the emergence of several clones associated with this serotype worldwide through capsule switching due to recombination-mediated swapping of the capsule locus between pneumococcal strains, as well as related streptococcal species [8]. However, detailed genomic analysis and phenotypic characterization of these serotype 3 lineages, especially those cocirculating in the same setting, have not been conducted thus far to determine why some of the serotype 3-associated lineages are more prevalent than others.

In the current study, we performed these genomic and phenotypic analyses to gain insights into the clonal expansion of the GPSC10-ST700 serotype 3 lineage in Malawi, the now-predominant serotype 3 lineage in Malawi, which is distinct from the CC180 lineage (GPSC12) more widespread globally [8]. We show that the GPSC10-ST700 serotype 3 lineage is common in Africa and has been identified among carriage and IPD isolates from Malawi and South Africa [17]. The serotype 3 strains in the GPSC10 lineage are characterized by the absence of 6 genes in the CPS locus, compared with the reference serotype 3 CPS locus sequence described by Bentley et al [24] and other serotype 3 lineages circulating in Malawi. The lineage is also tetracycline resistant, has a higher MIC for penicillin than other serotype 3 lineages isolated in Malawi, appears to have a propensity for capsule switching, and possesses virulence genes involved in pathogenicity and colonization. Furthermore, the GPSC10-ST700 lineage is less susceptible to opsonophagocytic killing than other serotype 3 lineages from Malawi carrying a full-length CPS locus, specifically ST5435 (GPSC9). Taken together, these characteristics may explain the apparent competitive advantage of the GPSC10-ST700 lineage compared with the other serotype 3 lineages identified after the introduction of PCV13 vaccine in Malawi [14, 16].

Serotype and genotype specific factors may have contributed to GPSC10-ST700 becoming the most prominent *S. pneumoniae* serotype 3 genotype in Malawi. It has been well documented that the PCV13 rollout has had minimal effect on serotype 3 carriage globally [9, 35, 37, 38], and this has been supported by post-PCV13 studies that have reported that PCV13 is less immunogenic for serotype 3 [4, 15, 39, 40]. Such evasion of vaccine-induced immunity could promote transmission of serotype 3 strains with certain virulence traits in both vaccinated and unvaccinated individuals, which may lead to the clonal expansion of serotype 3–associated lineages, including GPSC10-ST700. Together these factors may improve the population-level fitness and transmissibility of the GPSC10-ST700, especially following the elimination of competing vaccine type serotypes after PCV13 introduction. The reported increase of serotype 24F, associated with the GPSC10 genetic background after PCV13 introduction in France, further highlights the advantage of this genetic background [41].

The GPSC10-ST700 serotype 3 lineage appears to have emerged initially through capsular switching events, from serotype 19A/19F to serotype 3. Such acquisition of the serotype 3 capsule is not unexpected, as the GPSC10 genetic background is known to be highly permissive to capsule switching and expresses ≥16 serotypes [41]. This capsule switching has been associated with the acquisition of a shorter capsular locus that lacks the *wzg* (*cpsA*), *wzh* (*cpsB*), *wzd* (*cpsC*), and *wze* (*cpsD*) genes, also known as *cpsA-D* genes [24]. We propose that the absence of the genes in the GPSC-ST700 serotype 3 cps locus is more likely due to gene loss rather than to these genes having never been present, as this CPS locus structure was not widely evident before PCV13 introduction in Malawi or elsewhere. Indeed, there is increasing evidence supporting bacterial evolution through gene loss and genome reduction [42], including among *Streptococcus* species and between strains [43, 44]. It remains unknown whether this shorter version of the serotype 3 CPS locus has an impact on the structure of the serotype 3 capsule. However, we hypothesize that a shorter, yet functional serotype 3 CPS locus, may be acquired relatively easily by non–serotype 3 strains, including vaccine serotypes, leading to capsule switching. Further studies are required to fully understand the potential ecological benefit of the shorter serotype 3 CPS locus.

We show that the absence of the 6 genes in the GPSC10-ST700 serotype 3 CPS locus does not affect capsule production as detected by serotyping and colony morphology. We show that this lineage is less susceptible to opsonophagocytic killing with immune serum samples, which we postulate may be due to a modified capsule, contributing to immune evasion among PCV13-vaccinated individuals. Serotype 3, along with serotype 37, produces capsule using the synthase pathway rather than the Wzx/Wzy-dependent pathway [45–48]. As a result, only *wchE*, *ugd*, and *galU* are required [24]. However, we acknowledge that it is also possible that other genes coding subcapsular epitopes may contribute to the lower susceptibility in serum-mediated killing by ST700 compared with ST5435.

In conclusion, in the context of considerable global concern over the efficacy of current PCVs against serotype 3 [6, 7], we highlight the clonal expansion of a GPSC10-ST700 serotype 3 lineage after PCV13 introduction, which exhibits elevated penicillin MICs, possesses clusters of virulence genes involved in pathogenicity and colonization, is resistant to opsonophagocytosis, and has a propensity for capsule switching. These findings demonstrate the importance of continued genomic surveillance among vulnerable populations to improve pneumococcal vaccine effectiveness. This globally important pathogen is naturally competent, which may promote the acquisition of further AMR [48] and emergence of novel lineages through capsule switching. Therefore, we suggest that these characteristics are likely to enhance vaccine escape and should be considered when designing the next generation of pneumococcal vaccines. The *S. pneumoniae* serotype 3 human challenge model, which appears to be safe and achieves experimental colonization rates similar to those previously seen with *S. pneumoniae* serotype 6B [49], may offer the opportunity to better understand serotype 3 colonization and test the efficacy of novel vaccines.

## Supplementary Material

jiae040_Supplementary_Data

## Supplementary Data

Supplementary materials are available at *The Journal of Infectious Diseases* online (http://jid.oxfordjournals.org/). Supplementary materials consist of data provided by the author that are published to benefit the reader. The posted materials are not copyedited. The contents of all supplementary data are the sole responsibility of the authors. Questions or messages regarding errors should be addressed to the author.

## Notes

***Acknowledgments.*** We thank the individuals who participated in this study and the local schools and authorities for their support. We are grateful for the study field teams, the Malawi-Liverpool-Wellcome (MLW) laboratory team, and the hospitality of the QECH ART Clinic, led by Ken Malisita. Our thanks also extend to the MLW laboratory management team (led by Brigitte Denis) and the MLW data management team (led by the late Clemens Masesa). We thank Andrea Gori for his expert input into the analysis. Finally, we thank the High-Throughput Genomics Group at the Wellcome Trust Centre for Human Genetics (funded by Wellcome Trust grant 203141/Z/16/Z) for generation of the sequencing data.

***Disclaimer.*** The views expressed in this publication are those of the authors and not necessarily those of the National Institute for Health and Care Research or the Department of Health and Social Care.

***Financial support.*** This work was supported by the Bill & Melinda Gates Foundation (grant OPP1117653 to R. S. H.), the Medical Research Council (MRC grant MR/N023129/1 and grant MR/T016329/1 to J. M. C.), the National Institute for Health and Care Research (NIHR) (using UK Aid from the UK government; grant 16/136/46 to R. S. H.), the Wellcome Trust (strategic award 206545/Z/17/Z supporting the Malawi-Liverpool-Wellcome Clinical Research Programme), and the NIHR Global Health Research Unit on Mucosal Pathogens (using UK Aid from the UK government; support to A.K., T. D. S., N. F., and R. S. H.)
