# Supplementary material for: Clonal Expansion of a Streptococcus pneumoniae Serotype 3 Capsule Variant Sequence Type 700 With Enhanced Vaccine Escape Potential After 13-Valent Pneumococcal Conjugate Vaccine Introduction
Source: J Infect Dis. 2024 Mar 26;230(1):e189–98. doi: 10.1093/infdis/jiae040 (PMC11272040; doi:10.1093/infdis/jiae040)
Supplement: jiae040_Supplementary_Data [file jiae040_supplementary_data.zip › Supplementary Table 1.docx]

| **Pubmlst ID** | **isolate alias** | **collection_date** | **Serotype** | **ST** | **GPSC** |
| --- | --- | --- | --- | --- | --- |
| 145916 | WTCHG_379285_286104 | 22-Jun-15 | 3 | 5435 | 9 |
| 145984 | WTCHG_379286_228189 | 31-Jul-15 | 3 | 5435 | 9 |
| 146055 | WTCHG_379286_266102 | 05-Aug-15 | 3 | 5435 | 9 |
| 146143 | WTCHG_385771_225103 | 25-Nov-15 | 3 | 5435 | 9 |
| 146158 | WTCHG_385771_231103 | 03-Nov-15 | 3 | 5435 | 9 |
| 146556 | WTCHG_441196_239115 | 08-Jul-16 | 3 | 5435 | 9 |
| 146650 | WTCHG_441196_289192 | 27-May-16 | 3 | 5435 | 9 |
| 146652 | WTCHG_441196_290180 | 22-Jun-16 | 3 | NA | 9 |
| 146832 | WTCHG_441197_291130 | 09-Aug-16 | 3 | 5435 | 9 |
| 146864 | WTCHG_444990_212153 | 19-Dec-16 | 3 | 5435 | 9 |
| 147048 | WTCHG_444991_226122 | 16-Feb-17 | 3 | 5435 | 9 |
| 147055 | WTCHG_444991_230127 | 11-Apr-17 | 3 | 5435 | 9 |
| 147333 | WTCHG_585650_212144 | 06-Jun-18 | 3 | 5435 | 9 |
| 147353 | WTCHG_585650_223181 | 24-Apr-18 | 3 | 5435 | 9 |
| 147543 | WTCHG_585651_251136 | 14-Jul-17 | 3 | 5435 | 9 |
| 147568 | WTCHG_585651_264185 | 22-Jun-17 | 3 | 5435 | 9 |
| 147702 | WTCHG_598912_248101 | 25-Feb-16 | 3 | 5435 | 9 |
| 147714 | WTCHG_598912_255124 | 09-Feb-16 | 3 | 5435 | 9 |
| 147778 | WTCHG_598912_289191 | 19-Sep-17 | 3 | 5435 | 9 |
| 147943 | WTCHG_598913_283169 | 02-Feb-18 | 3 | 5435 | 9 |
| 148067 | WTCHG_769357_71855161 | 09-Jul-18 | 3 | 5435 | 9 |
| 148569 | WTCHG_777461_72945366 | 05-Feb-19 | 3 | 5435 | 9 |
| 145780 | WTCHG_379285_213101 | 02-Jul-15 | 3 | 700 | 10 |
| 145859 | WTCHG_379285_265104 | 26-Jun-15 | 3 | 700 | 10 |
| 145864 | WTCHG_379285_267101 | 02-Jul-15 | 3 | 700 | 10 |
| 145912 | WTCHG_379285_284104 | 16-Jul-15 | 3 | 700 | 10 |
| 146014 | WTCHG_379286_245189 | 07-Aug-15 | 3 | 700 | 10 |
| 146074 | WTCHG_379286_275189 | 05-Aug-15 | 3 | 700 | 10 |
| 146137 | WTCHG_385771_214103 | 05-Aug-15 | 3 | 700 | 10 |
| 146219 | WTCHG_385771_253104 | 04-Nov-15 | 3 | 700 | 10 |
| 146247 | WTCHG_385771_268186 | 24-Jul-15 | 3 | 700 | 10 |
| 146260 | WTCHG_385771_276186 | 01-Jul-15 | 3 | 700 | 10 |
| 146264 | WTCHG_385771_278186 | 24-Jun-15 | 3 | 700 | 10 |
| 146281 | WTCHG_385771_289186 | 02-Jul-15 | 3 | 700 | 10 |
| 146384 | WTCHG_441195_246126 | 22-Feb-16 | 3 | 700 | 10 |
| 146400 | WTCHG_441195_254125 | 10-Mar-16 | 3 | 700 | 10 |
| 146428 | WTCHG_441195_269147 | 15-Feb-16 | 3 | 700 | 10 |
| 146448 | WTCHG_441195_279122 | 25-Feb-16 | 3 | 700 | 10 |
| 146465 | WTCHG_441195_288188 | 28-Jan-16 | 3 | NA | 10 |
| 146497 | WTCHG_441196_208190 | 30-May-16 | 3 | 700 | 10 |
| 146545 | WTCHG_441196_232193 | 01-Apr-16 | 3 | NA | 10 |
| 146560 | WTCHG_441196_241111 | 31-May-16 | 3 | 700 | 10 |
| 146574 | WTCHG_441196_248195 | 23-May-16 | 3 | 700 | 10 |
| 146588 | WTCHG_441196_256101 | 05-Jul-16 | 3 | 700 | 10 |
| 146604 | WTCHG_441196_264112 | 01-Apr-16 | 3 | 700 | 10 |
| 146620 | WTCHG_441196_272186 | 11-Mar-16 | 3 | 700 | 10 |
| 146621 | WTCHG_441196_273103 | 17-Feb-16 | 3 | 700 | 10 |
| 146646 | WTCHG_441196_287176 | 23-Mar-16 | 3 | 700 | 10 |
| 146673 | WTCHG_441197_205142 | 13-Sep-16 | 3 | 700 | 10 |
| 146690 | WTCHG_441197_213156 | 21-Jul-16 | 3 | 700 | 10 |
| 146758 | WTCHG_441197_252160 | 02-Sep-16 | 3 | NA | 10 |
| 146862 | WTCHG_444990_211165 | 09-Dec-16 | 3 | NA | 10 |
| 146896 | WTCHG_444990_231115 | 01-Dec-16 | 3 | NA | 10 |
| 146916 | WTCHG_444990_243136 | 09-Jan-17 | 3 | 700 | 10 |
| 146922 | WTCHG_444990_246125 | 12-Oct-16 | 3 | 700 | 10 |
| 146962 | WTCHG_444990_274181 | 27-Jan-17 | 3 | 700 | 10 |
| 146975 | WTCHG_444990_283129 | 27-Sep-16 | 3 | NA | 10 |
| 146980 | WTCHG_444990_285153 | 30-Sep-16 | 3 | 700 | 10 |
| 146995 | WTCHG_444990_294131 | 13-Dec-16 | 3 | NA | 10 |
| 146997 | WTCHG_444990_295119 | 13-Dec-16 | 3 | 700 | 10 |
| 147023 | WTCHG_444991_212144 | 20-Feb-17 | 3 | 700 | 10 |
| 147059 | WTCHG_444991_232103 | 11-Apr-17 | 3 | NA | 10 |
| 147192 | WTCHG_585648_219132 | 05-Sep-17 | 3 | 700 | 10 |
| 147198 | WTCHG_585648_223180 | 26-Sep-17 | 3 | 700 | 10 |
| 147219 | WTCHG_585648_236146 | 12-Oct-17 | 3 | 700 | 10 |
| 147277 | WTCHG_585648_271174 | 10-Jul-17 | 3 | 700 | 10 |
| 147279 | WTCHG_585648_273103 | 13-Jul-17 | 3 | 700 | 10 |
| 147303 | WTCHG_585648_289105 | 06-Sep-17 | 3 | 700 | 10 |
| 147307 | WTCHG_585648_291129 | 02-Aug-17 | 3 | 700 | 10 |
| 147341 | WTCHG_585650_217188 | 19-May-17 | 3 | 700 | 10 |
| 147346 | WTCHG_585650_220145 | 24-Apr-18 | 3 | 700 | 10 |
| 147348 | WTCHG_585650_221140 | 08-Jan-16 | 3 | 700 | 10 |
| 147351 | WTCHG_585650_222169 | 13-Apr-18 | 3 | 700 | 10 |
| 147357 | WTCHG_585650_225187 | 05-Feb-16 | 3 | 700 | 10 |
| 147380 | WTCHG_585650_249196 | 10-Apr-18 | 3 | 700 | 10 |
| 147454 | WTCHG_585650_292142 | 19-Apr-18 | 3 | 700 | 10 |
| 147465 | WTCHG_585651_202118 | 22-Aug-17 | 3 | 700 | 10 |
| 147467 | WTCHG_585651_203130 | 07-Aug-17 | 3 | 700 | 10 |
| 147498 | WTCHG_585651_225109 | 17-Aug-17 | 3 | NA | 10 |
| 147506 | WTCHG_585651_230128 | 25-Sep-17 | 3 | NA | 10 |
| 147507 | WTCHG_585651_230169 | 04-Sep-17 | 3 | 700 | 10 |
| 147563 | WTCHG_585651_261148 | 23-Aug-17 | 3 | 700 | 10 |
| 147565 | WTCHG_585651_262161 | 09-May-17 | 3 | 700 | 10 |
| 147615 | WTCHG_585651_292156 | 04-Aug-17 | 3 | NA | 10 |
| 147642 | WTCHG_598912_213141 | 21-Dec-15 | 3 | 700 | 10 |
| 147645 | WTCHG_598912_215117 | 27-Jul-15 | 3 | 700 | 10 |
| 147655 | WTCHG_598912_220152 | 25-Jan-16 | 3 | NA | 10 |
| 147680 | WTCHG_598912_236146 | 06-Dec-17 | 3 | NA | 10 |
| 147723 | WTCHG_598912_260137 | 29-Feb-16 | 3 | 700 | 10 |
| 147737 | WTCHG_598912_267126 | 12-Jan-17 | 3 | 700 | 10 |
| 147753 | WTCHG_598912_275169 | 04-Mar-16 | 3 | 700 | 10 |
| 147758 | WTCHG_598912_279121 | 18-Aug-15 | 3 | 700 | 10 |
| 147768 | WTCHG_598912_284156 | 29-Mar-16 | 3 | 700 | 10 |
| 147775 | WTCHG_598912_288108 | 01-Sep-17 | 3 | NA | 10 |
| 147803 | WTCHG_598913_206131 | 13-Dec-17 | 3 | NA | 10 |
| 147871 | WTCHG_598913_244150 | 06-Feb-18 | 3 | 700 | 10 |
| 147917 | WTCHG_598913_270135 | 18-Jan-18 | 3 | 700 | 10 |
| 148045 | WTCHG_769357_71605136 | 27-Jul-18 | 3 | 700 | 10 |
| 148060 | WTCHG_769357_71765152 | 26-Jul-18 | 3 | 700 | 10 |
| 148072 | WTCHG_769357_71905166 | 09-Jul-18 | 3 | 700 | 10 |
| 148083 | WTCHG_769357_72035275 | 10-Aug-18 | 3 | 700 | 10 |
| 148180 | WTCHG_774610_70725048 | 09-Oct-18 | 3 | 700 | 10 |
| 148262 | WTCHG_774610_73055377 | 04-Oct-18 | 3 | 700 | 10 |
| 148274 | WTCHG_774610_73195295 | 03-Oct-18 | 3 | 700 | 10 |
| 148294 | WTCHG_774610_73465322 | 12-Oct-18 | 3 | 700 | 10 |
| 148331 | WTCHG_774611_70095081 | 03-Apr-19 | 3 | NA | 10 |
| 148410 | WTCHG_774611_72685244 | 03-Apr-19 | 3 | 700 | 10 |
| 148559 | WTCHG_777461_71865162 | 22-Feb-19 | 3 | NA | 10 |
| 148618 | WTCHG_777461_73505326 | 01-Feb-19 | 3 | 700 | 10 |
| 148626 | WTCHG_777461_73595335 | 11-Feb-19 | 3 | 700 | 10 |
| 148643 | WTCHG_777461_73795355 | 29-Jan-19 | 3 | 700 | 10 |
| 147185 | WTCHG_585648_214167 | 07-Jun-17 | 14 | 230 | 10 |
| 148439 | WTCHG_774611_73395315 | 15-Apr-19 | 14 | 230 | 10 |
| 147302 | WTCHG_585648_288188 | 29-Sep-17 | 10A | 3135 | 10 |
| 147911 | WTCHG_598913_267127 | 06-Apr-18 | 19A | NA | 10 |
| 146023 | WTCHG_379286_250102 | 07-Aug-15 | 19F | 10879 | 10 |
| 146061 | WTCHG_379286_269102 | 31-Jul-15 | 19F | 10879 | 10 |
| 146075 | WTCHG_379286_276102 | 04-Aug-15 | 19F | 10879 | 10 |
| 146083 | WTCHG_379286_280102 | 07-Aug-15 | 19F | 10879 | 10 |
| 146988 | WTCHG_444990_289191 | 07-Dec-16 | 19F | 10879 | 10 |
| 147151 | WTCHG_444991_290118 | 05-Apr-17 | 19F | 10879 | 10 |
| 147422 | WTCHG_585650_273193 | 29-May-18 | 19F | 10879 | 10 |
| 147564 | WTCHG_585651_262136 | 19-Jul-17 | 19F | 10879 | 10 |
| 147878 | WTCHG_598913_248196 | 08-May-18 | 19F | 10879 | 10 |
| 147903 | WTCHG_598913_263124 | 16-Feb-18 | 19F | 10879 | 10 |
| 148081 | WTCHG_769357_72015273 | 25-Jul-18 | 19F | 10879 | 10 |
| 148393 | WTCHG_774611_72485224 | 12-Mar-19 | 19F | 10879 | 10 |
| 148451 | WTCHG_774611_73525328 | 26-Mar-19 | 19F | 10879 | 10 |
| 145853 | WTCHG_379285_263104 | 23-Jun-15 | NT | 700 | 10 |
| 148632 | WTCHG_777461_73665342 | 13-Feb-19 | 3 | NA | 14 |
| 145838 | WTCHG_379285_257104 | 22-Jun-15 | 3 | 3214 | 43 |
| 145975 | WTCHG_379286_220104 | 31-Jul-15 | 3 | 3214 | 43 |
| 146048 | WTCHG_379286_262189 | 17-Nov-15 | 3 | 3214 | 43 |
| 146338 | WTCHG_441195_222129 | 10-Feb-16 | 3 | NA | 43 |
| 146676 | WTCHG_441197_206167 | 14-Jul-16 | 3 | 3214 | 43 |
| 147113 | WTCHG_444991_269146 | 01-Feb-17 | 3 | 3214 | 43 |
| 145997 | WTCHG_379286_236102 | 30-Jul-15 | 3 | 458 | 51 |
| 146777 | WTCHG_441197_262135 | 27-Sep-16 | 3 | 8672 | 56 |
| 146808 | WTCHG_441197_278133 | 22-Aug-16 | 3 | 8672 | 56 |
| 147068 | WTCHG_444991_237138 | 16-Feb-17 | 3 | 8672 | 56 |
| 147628 | WTCHG_598912_205154 | 05-Dec-17 | 3 | 8672 | 56 |
| 148106 | WTCHG_769357_72315207 | 14-Aug-18 | 3 | 11769 | 56 |
| 148506 | WTCHG_777461_71215097 | 30-Oct-18 | 3 | 8672 | 56 |
| 146804 | WTCHG_441197_276140 | 01-Aug-16 | 3 | 7105 | 92 |
| 147256 | WTCHG_585648_257196 | 08-May-17 | 3 | 10568 | 163 |
| 147728 | WTCHG_598912_262161 | 17-Feb-16 | 3 | 10568 | 163 |
| 147190 | WTCHG_585648_217189 | 10-May-17 | 3 | 9552 | 184 |
| 147592 | WTCHG_585651_278163 | 09-May-17 | 3 | 9552 | 184 |
| 147612 | WTCHG_585651_291129 | 10-May-17 | 3 | 9552 | 184 |
| 146928 | WTCHG_444990_249101 | 22-Sep-16 | 3 | NA | 455 |
| 145772 | WTCHG_379285_209101 | 24-Jun-15 | 3 | 6441 | 648 |
| 146058 | WTCHG_379286_267189 | 29-Jul-15 | 3 | NA | NA |
| 147512 | WTCHG_585651_233110 | 15-Aug-17 | 3 | 11770 | NA |
