# Supplementary material for: Clonal Expansion of a Streptococcus pneumoniae Serotype 3 Capsule Variant Sequence Type 700 With Enhanced Vaccine Escape Potential After 13-Valent Pneumococcal Conjugate Vaccine Introduction
Source: J Infect Dis. 2024 Mar 26;230(1):e189–98. doi: 10.1093/infdis/jiae040 (PMC11272040; doi:10.1093/infdis/jiae040)
Supplement: jiae040_Supplementary_Data [file jiae040_supplementary_data.zip › Supplementary table legends.docx]

**SUPPLEMENTARY MATERIAL – Table Legends**

**Table S1: Isolates analysed in this study and associated metadata.**

**Table S2: cps sequences of representative serotype 3 sequence types.**
